# Supplementary material for: New insight into the significance of KLF4 PARylation in genome stability, carcinogenesis, and therapy
Source: EMBO Mol Med. 2020 Nov 24;12(12):e12391. doi: 10.15252/emmm.202012391 (PMC7721363; doi:10.15252/emmm.202012391)
Supplement: Supplementary file 1 — Appendix [file EMMM-12-e12391-s001.pdf]

Appendix information

Table of content:

1. Appendix Figure S1 and figure legend

2. Appendix Figure S2 and figure legend

3. Appendix Figure S3 and figure legend

4. Appendix Figure S4 and figure legend

5. Appendix Figure S5 and figure legend

6. Appendix Figure S6 and figure legend

7. Appendix Figure S7 and figure legend

8. Appendix Figure S8 and figure legend

9. Appendix Table S1

10. Appendix Table S2

11. Appendix Table S3

12. Appendix Table S4 (exact p-values)

1. Appendix Figure S1 and figure legend

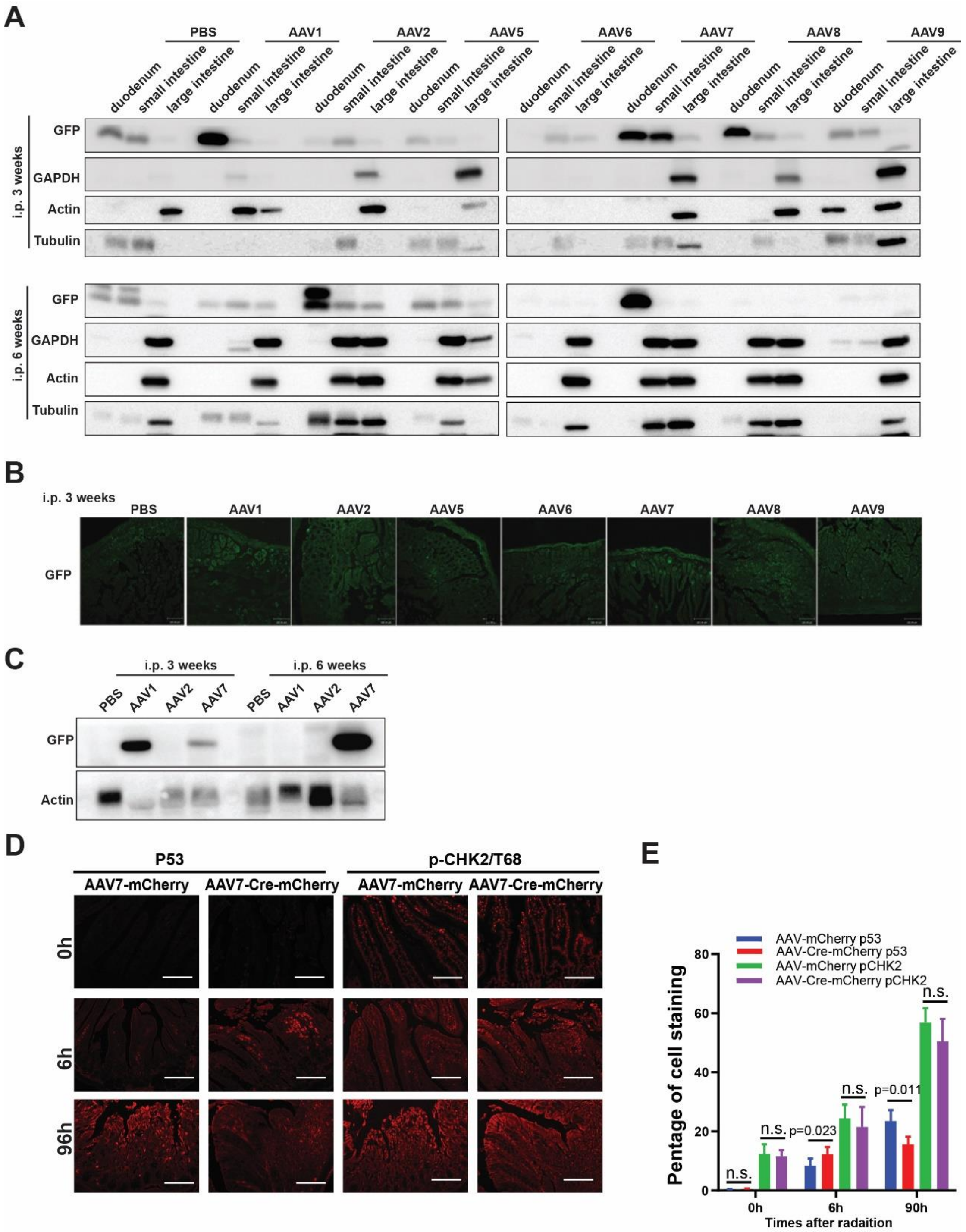

Figure S1. Establishment of KLF4 $^{loxp/loxp}$/ AAV-Cre-mCherry conditional adult gut-intestine knockout mice model. (**A**) AAV7 pseudotype exhibit the strongest intestinal tissue tropism than that of other AAV pseudotypes (AAV1, AAV2, AAV5, AAV6, AAV8 and AAV9). The tissue tropism of AAV serotypes 1-9 which harbor GFP gene were analyzed via intraperitoneal injection. $2x10^{11}$ particles of AAV1, AAV2, AAV5, AAV6, AAV7, AAV8 and AAV9 were intraperitoneally injected into KLF4 $^{loxp/loxp}$ mice (6~8 weeks), respectively. Three weeks later, the expression of GFP in duodenum, small intestine and large intestine were detected by Western blotting. GAPDH, Actin and Tubulin are loaded as control. (**B**)

The expression of GFP in the duodenum. $2x10^{11}$ particles of AAV1, AAV2, AAV5, AAV6, AAV7, AAV8 and AAV9 that harbor GFP gene were intraperitoneally injected into KLF4$^{loxp/loxp}$ mice, three days after the injection, the duodenum was collected and sectioned followed by measurements of GFP expression by fluorescence microscopy. Scale bars, 100 μm. (**C**) Intraperitoneal injection of AAV7 pseudotype allows GFP expression for 6 weeks. AAV1, AAV2 and AAV7 were intraperitoneally injected into KLF4$^{loxp/loxp}$ mice and the expression of GFP in duodenums were then detected by Western blotting at three weeks and six weeks after AAV administration. (**D & E**) Immunofluorescent staining of p53 and p-CHK2/Thr68 in the intestinal epithelium of KLF4 $^{loxp/loxp}$ mice with injection of AAV7-mCherry or AAV7-Cre-mCherry followed by treatment of irradiation. Tissues were collected from sham mice and mice at different time after exposure to irradiation and then staining with indicated antibodies. (**E**) Quantification of p53 and p-CHK2/Thr68 based on the Immunofluorescent staining results presented in **D**. Data are mean ± SEM; n=3, *p* values were indicated in figures, one-way ANOVA assay. Scale bars, 60 μm. No difference of p-CHK2 at different time point between AAV7-mCherry and AAV7-Cre-mCherry group. P53 seems lower in 6h but higher at 96h after radiation in AAV7-Cre-mCherry group, which is not consistent.

Appendix Figure S2 and figure legend

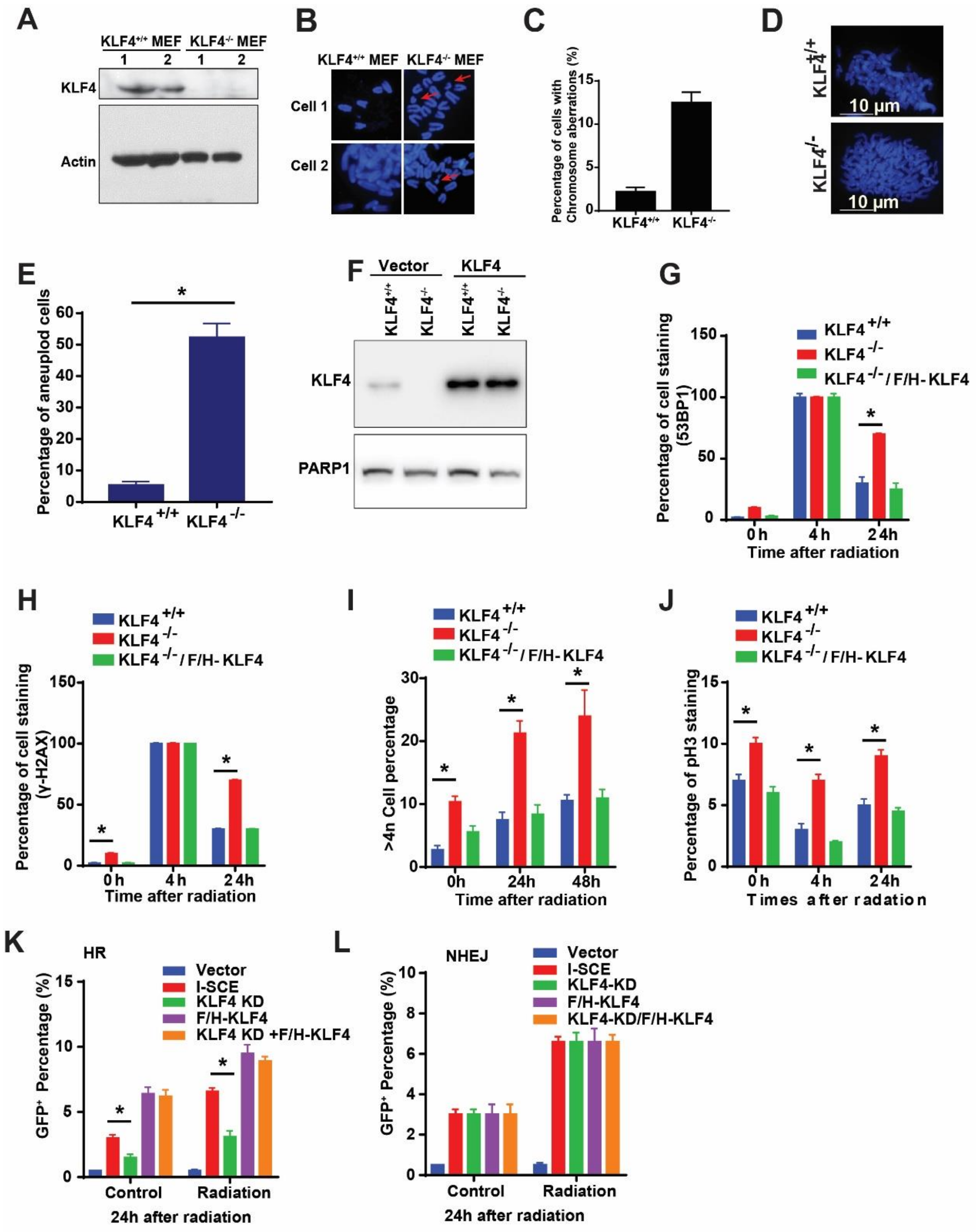

Figure S2 KLF4 orchestrates DNA damage response and DNA repair. (**A**) Measurement of KLF4 expression in KLF4 knockout MEFs. (**B &C**) Aberrations of KLF4 leads to chromosome breaks. n=20, *p*=, one-way ANOVA assay. (**D &E**) Cytogenetic analysis of ploidy in $KLF4^{+/+}$ and $KLF4^{-/-}$ MEFs. Karyotype analysis was conducted in metaphase chromosome spreads prepared from $KLF4^{+/+}$ and $KLF4^{-/-}$ MEFs. n=20, *p*=, one-way ANOVA assay. Scale bars, 10 μm. (**F**) Expression of KLF4 and PARP1 in KLF4 knockout MEFs and $KLF4^{-/-}$ MEFs with added back expression of KLF4. (**G**) Expression of 53BP1 foci in $KLF4^{+/+}$ and $KLF4^{-/-}$ MEFs at 6h or 24h after

5Gy radiation. Loss of KLF4 causes retardation of 53BP1 foci resolution after exposure to DNA damage signal. Reconstitution of KLF4 recovers the efficiency of 53BP1 foci resolution in $KLF4^{-/-}$ MEFs. n=3, *p* values were indicated in figures, one-way ANOVA assay. (**H**) Expression of γ-H2AX in $KLF4^{+/+}$ and $KLF4^{-/-}$ MEFs at 6h or 24h after 5Gy radiation. Loss of KLF4 caused retardation of γ-H2AX foci resolution after DNA damage. Reconstitution of KLF4 restores the efficiency of γ-H2AX foci resolution in $KLF4^{-/-}$ MEFs. n=3, *p* values were indicated in figures, one-way ANOVA assay. (**I-J**) Loss of KLF4 in MEFs cells results in increased aneuploidy cells (>4n) and pH3 positive cells. Loss of KLF4 also retards DNA damage response. More pH3 positive cells and aneuploidy cells were observed in $KLF4^{-/-}$ cells at 24h and 48h after DNA damage in comparison to $KLF4^{+/+}$ MEF cells. Addback KLF4 in $KLF4^{-/-}$ MEFs recovers the phenotype. n=3, *p* values were indicated in figures, one-way ANOVA assay. (**I**) The summary of >4n cells distribution. n=3, *p* values were indicated in figures, one-way ANOVA assay. (**J**) The summary of pH3 positive cell distribution. n=3, *p* values were indicated in figures, one-way ANOVA assay. (**K-L**). HR (Homologous recombination) and NHEJ (Non-homologous end join) assay in U2Os-GFP-EJ5 cells. Control indicates without radiation. Radiation indicates 5Gy radiation. (**K**) HR assay. Knockdown KLF4 in U2OS decreases HR, while overexpressed KLF4 increases HR. (**L**) NHEJ assay. Modulation of KLF4 expression has no effect on NHEJ efficacy. n=3, *p* values were indicated in figures, one-way ANOVA assay. The exact p-Values were supplied in Appendix Table S4.

Appendix Figure S3 and figure legend

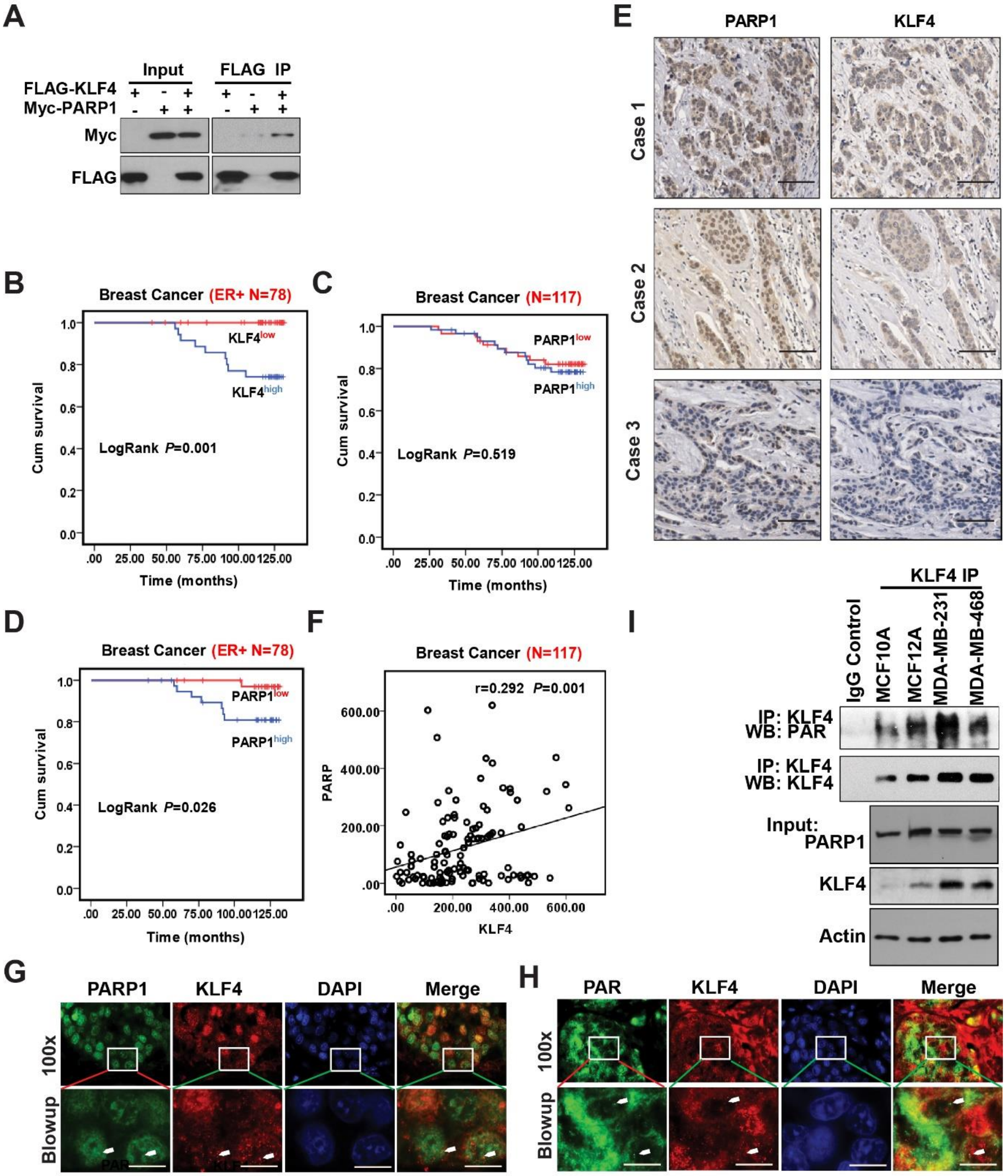

Figure S3. Interaction between KLF4 and PARP1 in breast cancer cells and expression of KLF4 and PARP1 in human breast cancer specimens. (**A**) Ectopic KLF4 and PARP1 co-immunoprecipitated in breast cancer cells MDA-MB-231. (**B**) Survival analysis of KLF4 protein expression in 78 ER positive breast cancer patient. Compared to patients with low KLF4 protein expression, patients with high protein expression had an inferior cumulative survival rate (Kaplan–Meier assay, LogRank P=0.001). (**C**) Survival analysis of PARP1 protein expression in 117 breast cancer patients. No significant difference in cumulative survival rate between patient group with high PARP1 protein expression and patient group

with low PARP1 protein expression (Kaplan–Meier assay, LogRank P=0.519). (**D**) Survival analysis of PARP1 protein expression in 78 ER positive breast cancer patient. Compared to patients with low PARP1 protein expression, patients with high protein expression had an inferior cumulative survival rate (Kaplan–Meier assay, LogRank P=0.026). (**E & F**) Elevated expression of PARP1 and KLF4 are significantly correlated in 117 cases of human breast cancer tissue specimens (Tissue array XT16-054). Represents of paired IHC staining of PARP1 and KLF4 (case 1-3) are shown (**E**). Scale bars, 100 μm. (**F**) Statistic analysis of IHC staining in 117 cases of breast cancer patient which contains prognosis data indicates that PARP1 expression is positively correlated with KLF4 expression in breast cancer (Tissue array XT16-054) (r=0.292, n=117, *p*=0.001, Pearson correlation coefficients). (**G & H**) Measurement of KLF4 PARylation. (**H**) PARP1 and KLF4 are colocalized in breast cancer tissue. Scale bars, 10 μm. (**I**) PARylation of KLF4 is detected by using antibody against PAR. Scale bars, 10 μm). (**I**). KLF4 PARylation is highly prone to accumulate in MDA-MB-231, MDA-MB-468 in comparison to normal mammary gland epithelial cell line MCF10A and MCF12A.

Appendix Figure S4 and figure legend

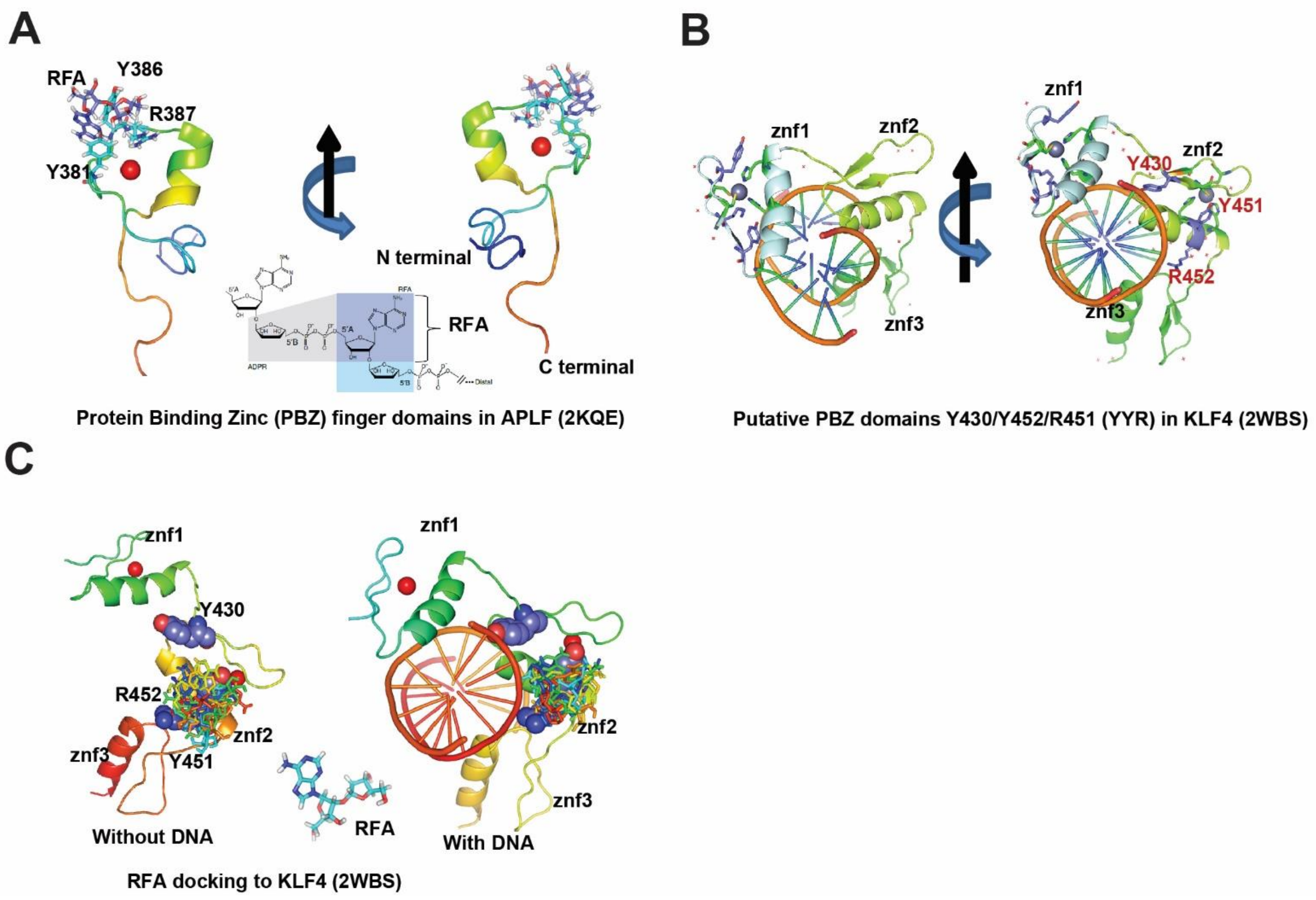

Figure S4. Results on KLF PARylation site from structure-based simulations of KLF4- PARP1 interactions (A) Protein Binding Zinc (PBZ) domains in APLF (2KQE). The RFA (2'-O-α-D-ribofuranosyl adenosine) binds the zinc finger motif containing the YYR motif formed by Y386, Y381 and R387. (**B**) The PBZ domain Y430/Y451/R452 motif (YYR) in KLF4 (PDB id: 2WBS) is structurally comparable to that in APFL. (**C**) Docking of RFA onto the YYR motif of KLF4. *Left panel*, without DNA; *Right panel*, with DNA.

Appendix Figure S5 and figure legend

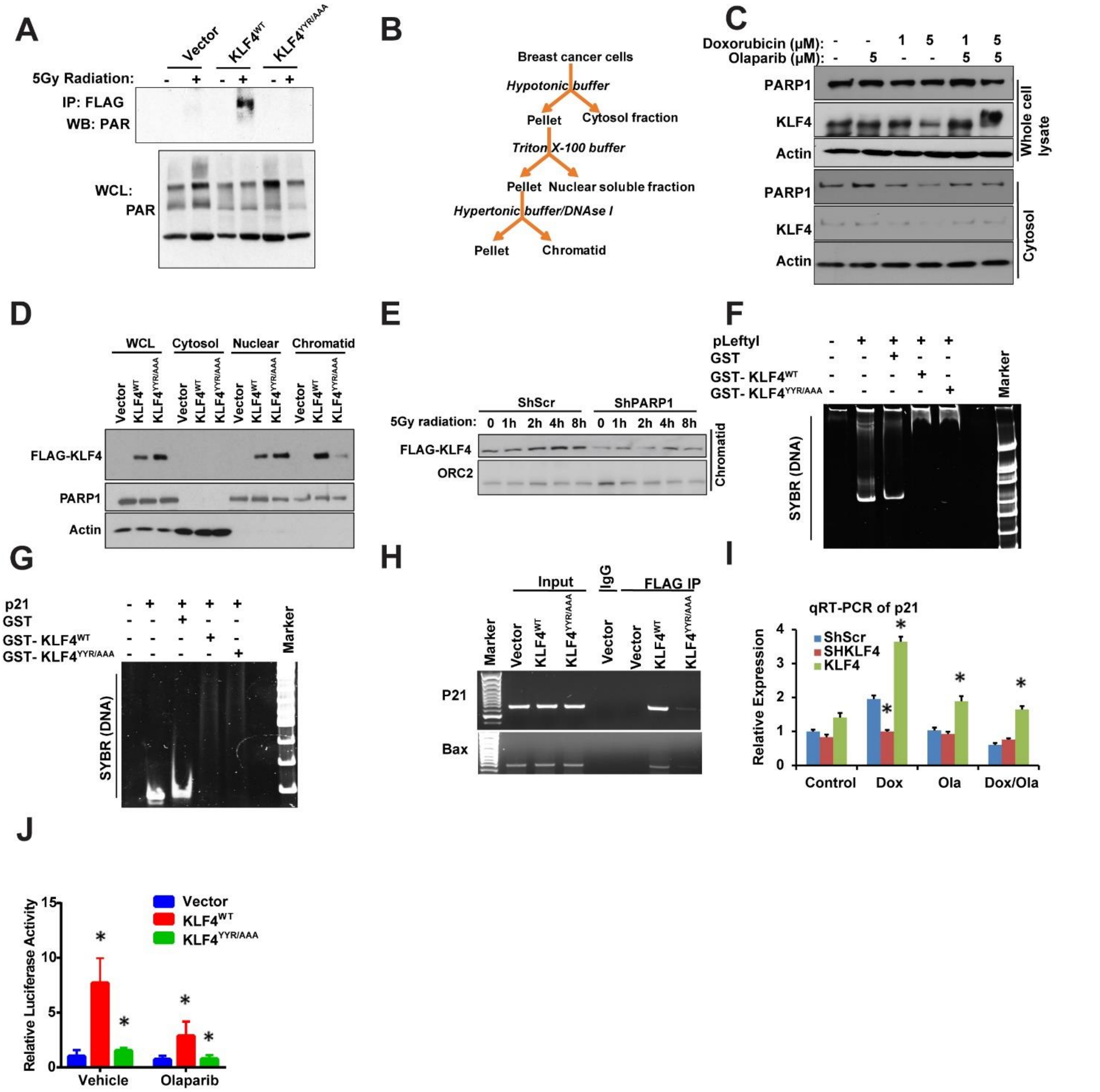

Figure S5. Mechanistic role of DNA damage-induced KLF4 PARylation in orchestrating the recruitment of KLF4 to the chromatin. (**A**) Replacement of KLF4 YYR motif residues by three alanines abrogates KLF4 PARylation. Wild-type or YYR/AAA mutant KLF4 were transfected into 293T cells followed by treatment with 5Gy radiation 48 hrs after the transfection. 4 hrs after radiation, the cells were lysed and the FLAG tagged KLF4 was pulled down by M2 beads followed by detecting KLF4 PARylation using antibody against PAR. (**B**) The schematic flow-chart showing the process for subcellular fraction of cytosol, nuclear soluble and chromatid extract. (**C**) Measurement of the effect of PARP1 inhibitor on the distribution of KLF4 among cytosol, soluble nucleus and chromatin. Both cytosol fraction and whole cell lysates were used for the measurement in MDA-MB-231 cells. (**D**) Distribution of ectopic expression wild-type ($KLF4^{WT}$) or mutant KLF4 ($KLF4^{YYR/AAA}$) in cytosol, nuclear soluble and

chromatid fraction. The KLF4$^{YYR/AAA}$ mutation decreases its chromatid location in MDA-MB-231 cells. (**E**) Knockdown PARP1 decreases the FLAG-KLF4 expression in chromatid fraction. (**F &G**) Mutation of KLF4 YYR motif does not affect KLF4 binding to promoter DNA of pLeftyl (**F**) and p21 (**G**). The purified GST-KLF4^{WT} or GST-KLF4$^{YYR/AAA}$ were subjected to electrophoretic mobility shift assay (EMSA assay) for pLeftyl and p21 promoter. The mobility shift of for pLeftyl and p21 promoter were detected by SYBR. (**H**) Validation of the impact of KLF4 PARylation on KLF4-mediated transcription using p21 and Bax promoter luciferase assay in 293T cells. Mutation of KLF4 PARylation decreases KLF4 binding to p21 and Bax promoter. (**I**) qRT-PCR analysis. Effect of KLF4 expression levels and Olaparib treatment on the expression of p21. Knockdown KLF4 or Olaparib treatment decrease expression of p21 in MDA-MB-231 cells. n=3, *p* values were indicated in figures, one-way ANOVA assay. (**J**) Validation of the impact of KLF4 PARylation on KLF4-mediated transcription by p21 promoter luciferase assay in 293T cells. The asterisk in the panels represents the significant difference ($p<0.05$). Data are mean ± SEM; n=3, the exact p-Values were supplied in Appendix Table S4, one-way ANOVA assay.

Appendix Figure S6 and figure legend

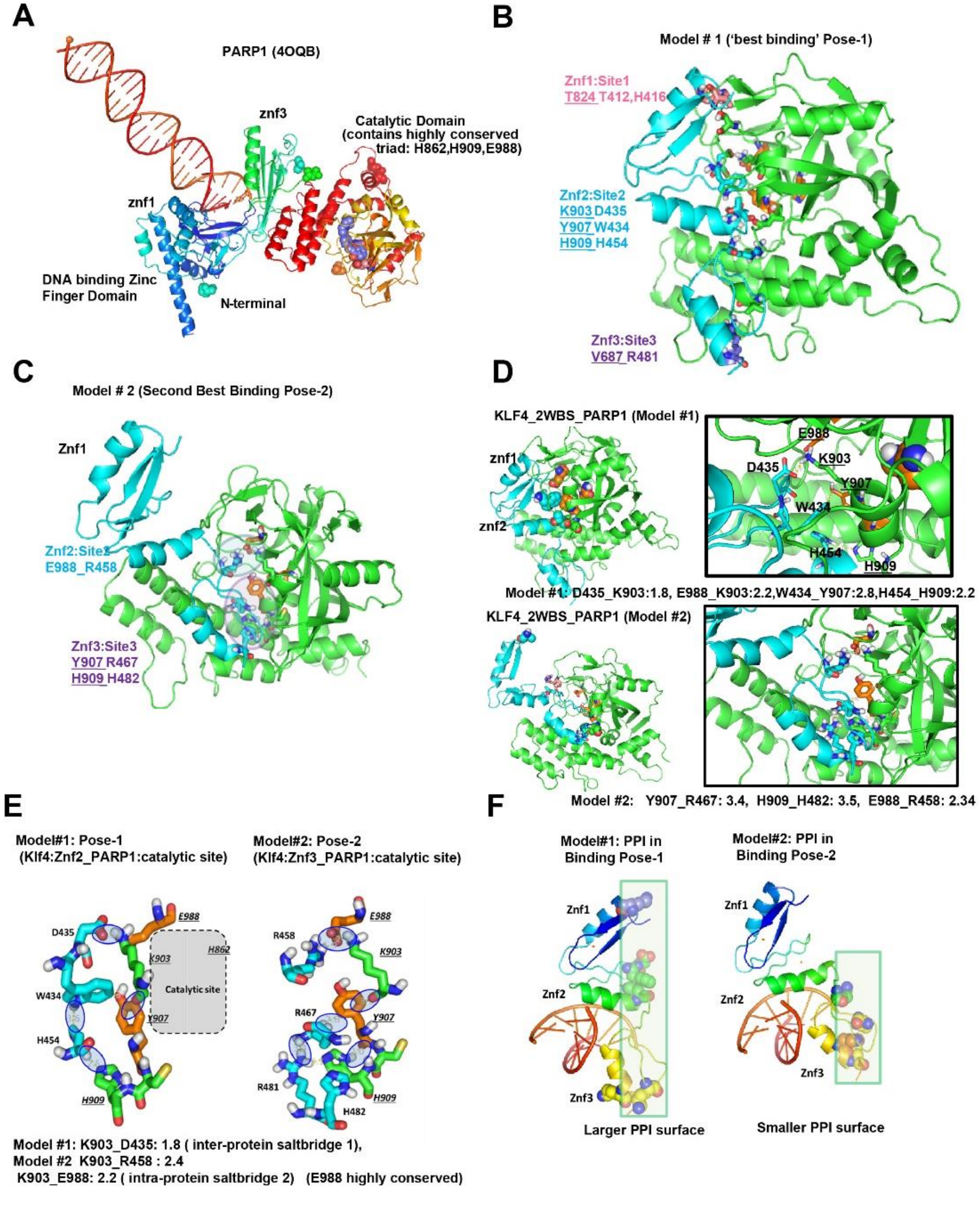

Figure S6. Molecular docking analysis of the interaction between the catalytic core of PARP1 and the KLF4 Zinc motifs. (**A**) The structure of DNA-Zinc motifs (Znf1, Znf2 and Znf3) and catalytic core of PARP1 (PDB id: 4OQB). The catalytic core of PARP1 contains a highly conserved triad: H862, H909 and E988. PARP1 also has three zinc fingers, of which Znf1 and Znf2 are involved in DNA

binding and Znf3 is not. (**B**) Model #1 obtained for PARP1-KLF4 interaction from docking simulations. Model #1 was the best (top-scoring) binding pose. Residue pairs that make intermolecular contacts are shown in *stick representation* and *labeled*. The underlined residues belong to PARP1 znf1, 2 and 3; and the KLF4 residues making close contacts with znf1-3 residues are listed in each case (not underlined). (**C**) Model #2 for KLF4-PARP1 binding, which is second best-scoring pose observed in docking simulations. Same nomenclature as in (**C**) for PARP1-KLF4 residue pairs making close contacts. (**D**) Comparison of Models #1 and #2. Closest distances between interfacial salt-bridge forming amino acids are indicated. Model #1, D435 (KLF4)-K903 (PARP1):1.8 Å, W434 (KLF4)-Y907 (PARP1):2.8 Å, and H454 (KLF4)-H909 (PARP1):2.2 Å. Model #2: Y907-R467: 3.4 Å, H909 (PARP1)-H482 (KLF4): 3.5 Å, and E988 (PARP1) -R458 (KLF4): 2.34 Å. Note that PARP1 residues E988 and K903 make an intramolecular salt bridge (closest atom-atom contacts of 2.2 Å), (**E**) The network of residue interactions in Model #1 (KLF4:Znf2_PARP1: catalytic site) and in Model #2 (KLF4:Znf3_PARP1: catalytic site). (**F**) Comparison of the Protein-Protein Interaction (PPI) Surface in Model #1 (large PPI surface) and Model #2 (small PPI surface) is in support of Model #1.

Appendix Figure S7 and figure legend

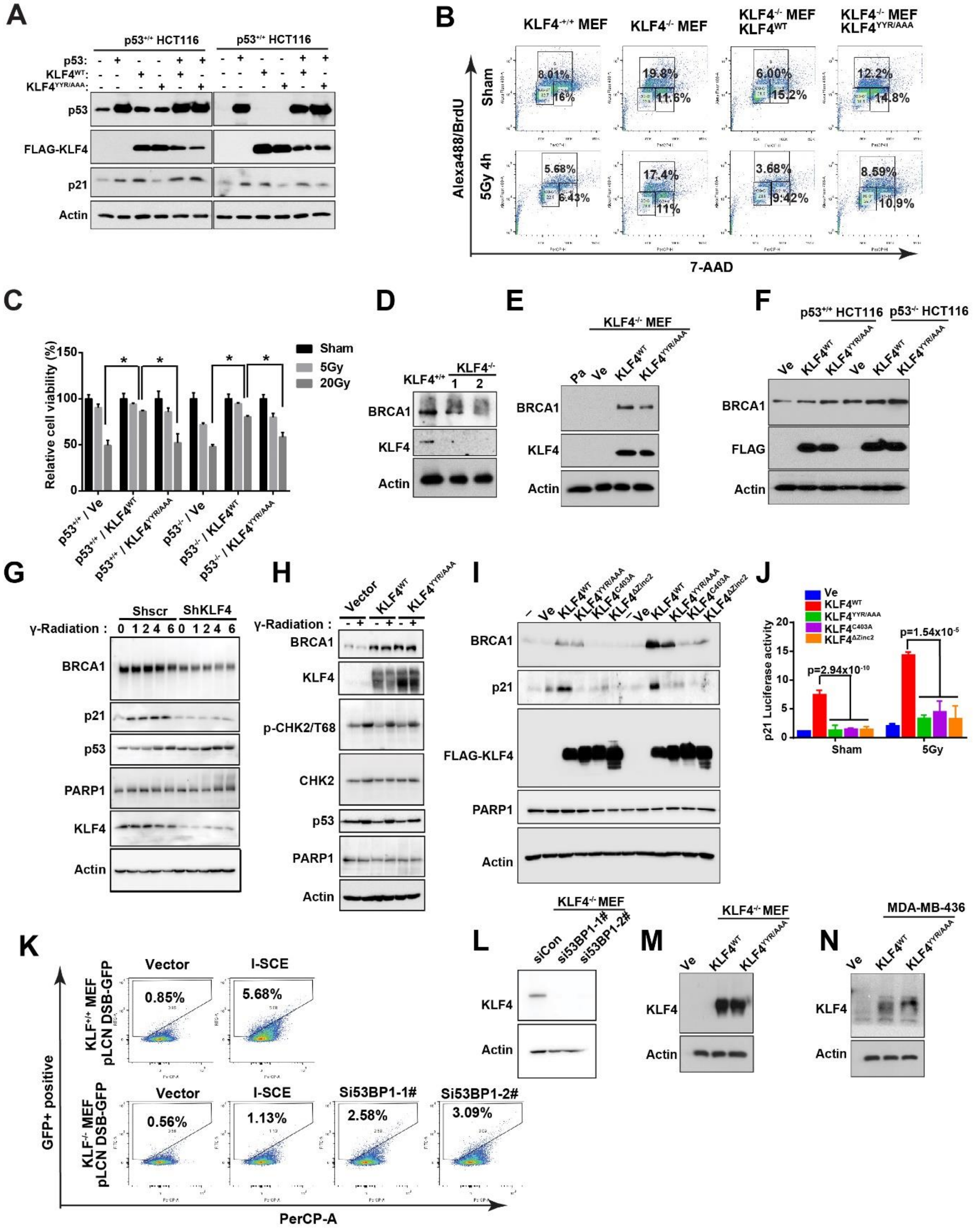

Figure S7. KLF4 regulates BRCA1 expression. (**A**) PARylation of KLF4 promotes p21 expression is independent on p53. $p53^{+/+}$ or $p53^{-/-}$ HCT116 cells were transfected with p53, wild-type ($KLF4^{WT}$) or mutant KLF4 ($KLF4^{YYR/AAA}$). Lysate were collected for detecting the expression of p21 and KLF4 48 hrs after the transfection. (**B**) BrdU chase assay. $KLF4^{+/+}$, $KLF4^{-/-}$, $KLF4^{-/-}/KLF4^{WT}$, $KLF4^{-/-}/KLF4^{YYR/AAA}$ with

5Gy irradiation 4h treatment and then chase with BrdU for 2 hrs, then staining with Alexa488-anti-BrdU and 7-AAD. KLF4$^{-/-}$ and KLF4$^{-/-}$/KLF4$^{YYR/AAA}$ show more S phase in compare with KLF4$^{+/+}$, KLF4$^{-/-}$/KLF4^{WT}. (**C**) p53. p53$^{+/+}$ or p53$^{-/-}$ HCT116 cells were transfected with p53, wild-type (KLF4^{WT}) or mutant KLF4 (KLF4$^{YYR/AAA}$) were treated with 5Gy or 20Gy radiation, and 48h later the cell viability were measured by CCK-8. KLF4^{WT} suppress cell death independent with p53. n=3, *p* values were indicated in figures, one-way ANOVA assay. **(D**) Expression of BRCA1 in both KLF4$^{+/+}$ and KLF4$^{-/-}$ MEFs. The lane 1 and 2 represent different passages of MEFs. Loss of KLF4 decrease BRCA1 expression. (**E**) Expression of BRCA1 in KLF4$^{-/-}$ MEFs with overexpression of wild-type or mutant KLF4. Both wildtype and mutant KLF4 increase BRCA1 expression. The KLF4$^{-/-}$ MEFs were transfected wildtype (KLF4^{WT}) or mutant KLF4 (KLF4$^{YYR/AAA}$). Lysates were collected for detecting the expression of BRCA1 and KLF4 48 hrs after the transfection. Pa, parental cells; Ve, Vector. (**F**) KLF4 promotes BRCA1 in both p53 wild-type or mutant cells. p53$^{+/+}$ or p53$^{-/-}$ HCT116 cells were transfected with wild-type (KLF4^{WT}) or mutant KLF4 (KLF4$^{YYR/AAA}$). Lysates were collected for detecting the expression of BRCA1 and KLF4. (**G**) MDA-MB-231-ShScr and ShKLF4 cells were treated with 5Gy radiation and the cell lysates were collected at indicated time. BRCA1, p21, p53, PARP1, KLF4 and actin were blotted. Knockdown KLF4 decrease BRCA1 and KLF4 expression, but slightly increase p53 expression. (**H**) U2OS cells were transfected with KLF4^{WT} or KLF4$^{YYR/AAA}$ plasmids and then subjected for γ-radiation treatment after 48h. Cell lysates were collected at 4h after radiation, and then blotting with BRCA1, KLF4, p-CHK2/Thr68, CHK2, P53, PARP1 and Actin. No difference of BRCA1, CHK2, p-CHK2/Thr, p53 between wildtype and mutant KLF4 (KLF4$^{YYR/AAA}$). (**H**) The expression of BRCA1 and p21 in KLF4$^{-/-}$ MEFs with transfection of wildtype (KLF4^{WT}) or mutant KLF4 (KLF4$^{YYR/AAA}$, KLF4^{C403A} and KLF4$^{\Delta Zinc2}$). While the wildtype (KLF4^{WT}) or mutant KLF4 (KLF4$^{YYR/AAA}$) could promote BRCA1 expression, only the wildtype (KLF4^{WT}) could enhance p21 expression. (**I**) PARylation of KLF4 promotes p21 expression is independent on p53. p53$^{+/+}$ or p53$^{-/-}$ HCT116 cells were transfected with p53, wild-type (KLF4^{WT}) or mutant KLF4 (KLF4$^{YYR/AAA}$). Lysate were collected for detecting the expression of p21 and KLF4 48 hrs after the transfection. (**J**) The p21 luciferase activity assay in KLF4$^{-/-}$ MEFs with transfection of wildtype (KLF4^{WT}) or mutant KLF4 (KLF4$^{YYR/AAA}$, KLF4^{C403A} and KLF4$^{\Delta Zinc2}$). Only the wildtype KLF4 could drive p21 promoter transcription. Data are mean ± SEM; n=3, *p* values were indicated in figures, one-way ANOVA assay. (**K&L**) KLF4$^{+/+}$ and KLF4$^{-/-}$cells were transfected with pLCN DSB Repair Reporter (DRR) for homologous recombination assay. KLF4$^{-/-}$-pLCN DSB-GFP cells were transfected with 53BP1 siRNA and then transfected with I-SCE and subjected to flow cytometry for GFP positive cells. (**K**) shows flow cytometry assay, (**L**) shows the western blotting of 53BP1 after siRNA transfected. (**M & N**) Expression of wildtype and mutant KLF4 in KLF4$^{-/-}$ MEFs (**M**) or MDA-MB-436 (**N**).

Appendix Figure S8 and figure legend

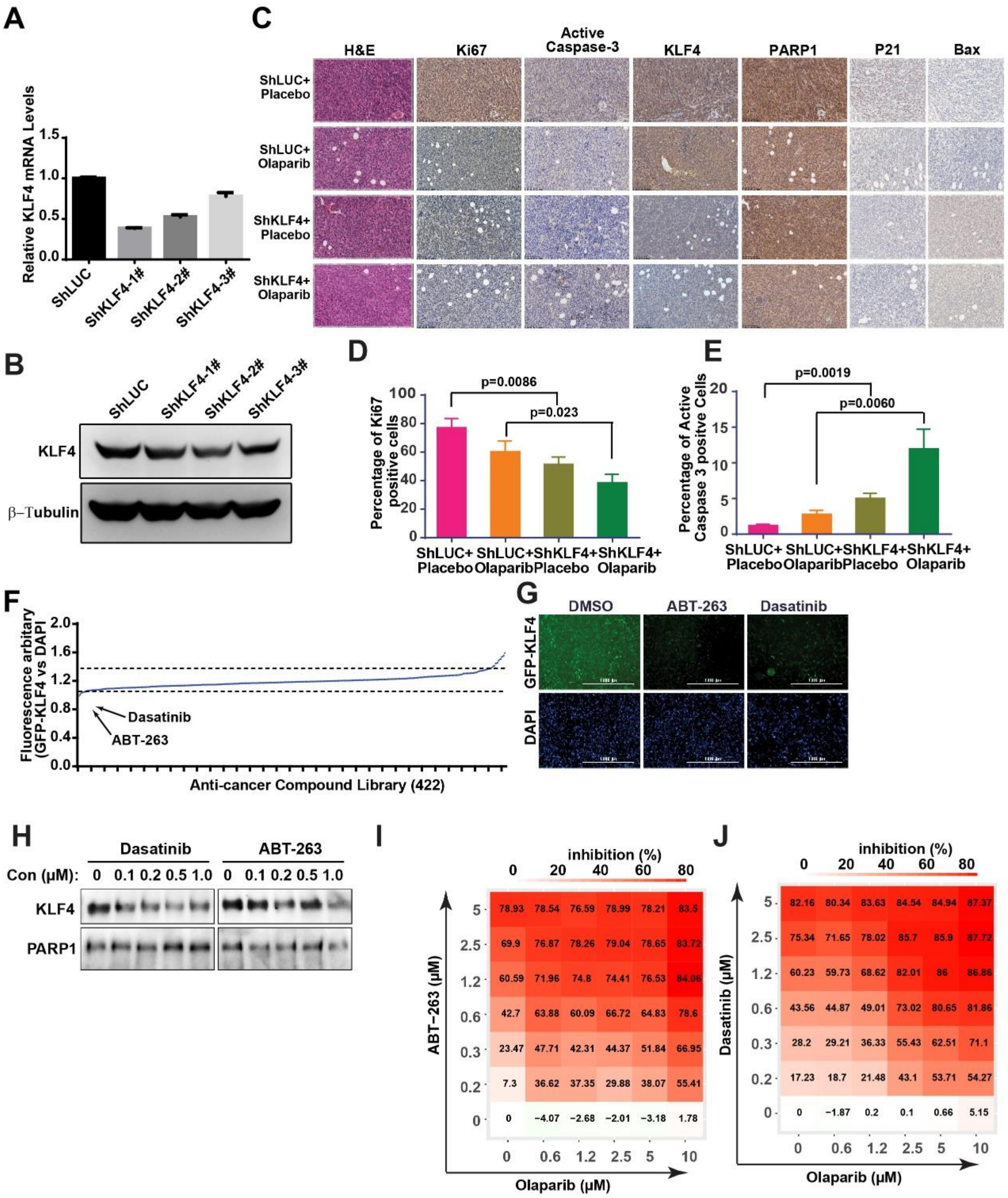

Figure S8. Expression of KLF4 in 4T1 cells and xenograft tumors. (**A**) Quantitative PCR assay of KLF4 mRNA expression in 4T1 cells. The ShKLF4-1# and ShKLF4-2# show about a 50% decrease of KLF4 mRNA. n=3, p=1.76x10^{-8}, one-way ANOVA assay. (**B**) Western blotting assay of KLF4 protein expression in 4T1 cells. The β-tubulin serves as the loading control. The 4T1 ShKLF4-2# was further used in the xenograft mice model. (**C**) Staining of H & E, Ki67, KLF4, PARP1, p21, Bax and activated caspase 3 in 4T1 xenograft tumors. Scale bars, 100 μm. Combination of KLF4 knockdown and Olaparib treatment decrease Ki67 positive population cells, decrease p21, but increase Bax and activated caspase-3 in xenograft tumors. (**D & E**) Enhanced efficacy of Olaparib in killing TNBC cells is due to inhibiting tumor cell proliferation (Ki67) and stimulating tumor cell for

apoptosis (active caspase 3). Summary of Ki67 (**D**) and active caspase 3 (**E**) staining. n=4, *p* values were indicated in figures, one-way ANOVA assay. (**F**) MDA-MB-231 with pLenti-GFP-KLF4 stable expression cells were treated with an anti-cancer compound (10 μM) for 24 hours and then the GFP and DAPI fluorescence strength was assayed. The rankings of the GFP fluorescence strength of 422 anti-cancer compounds library are shown by the plot map; each dot represents 1 anti-cancer compound. The top 2 anti-cancer compounds are listed. (**G**) Represent figures of MDA-MB-231-GFP-KLF4 expression after treated with AF-263 or dasatinib for 24h. Scale bars, 100 μm. (**H**) The western blot of endogenous KLF4 protein after treated with AF-263 or dasatinib at indicated concentration for 24h in MDA-MB-231 cells. (**I & J**) The combination effect of Olaparib with ABT-263 (**I**) and dasatinib (**J**). MDA-MB-231 cells were treated with combination dosage of Olaparib, ABT-263 (**I**) and dasatinib (**J**) at indicated dose for 24h. Cell survival was measured by CCK-8.

Appendix Table S1

Peptides of PARP1 (P09874) identified from mass spectrometry

Valid	...	Sequence	Prob	Mas...	Mas...	Mas...	NTT	Modifications	Observed	Actual M...	Char...	Delt...	Delt...	Rete...	Intensity	TIC	Start	Stop	# Ot...	Other Pr...	Spectrum ID
☑	✓	(R)MAIMVQSPMFDGK(V)	100%	62.3	41.3	43.0	2	Oxidation (+16...	752.31	1,502.61	2	0.94	-39	3630		0.0000	35	47	0		File:P073910....
☑	✓	(R)MAIMVQSPMFDGK(V)	92%	41.6	41.3	19.2	2	Oxidation (+16...	752.74	1,503.47	2	1.8	-130	3670		0.0000	35	47	0		File:P073910....
☑	✓	(K)TAEAGGVTGK(G)	100%	62.1	42.6	29.9	2		446.11	890.21	2	0.75	-280	948		0.0000	88	97	0		File:P073910....
☑	✓	(K)TAEAGGVTGK(G)	94%	43.2	42.4	18.3	2		446.18	890.35	2	0.89	-120	2050		0.0000	88	97	0		File:P073910....
☑	✓	(K)TLGDFAAEYAK(S)	100%	70.7	40.8	59.1	2		593.47	1,184.93	2	0.35	300	4310		0.0000	109	119	0		File:P073910....
☑	✓	(K)GFSLLATEDK(E)	98%	51.6	41.3	29.0	2		541.22	1,080.43	2	0.88	-120	4440		0.0000	183	192	0		File:P073910....
☑	✓	(K)QQVPSGESAILDR(V)	100%	65.6	41.3	50.6	2		700.45	1,398.89	2	0.17	120	3650		0.0000	270	282	0		File:P073910....
☑	✓	(K)QQVPSGESAILDR(V)	100%	62.4	41.4	45.2	2		700.71	1,399.41	2	0.69	-220	3720		0.0000	270	282	0		File:P073910....
☑	✓	(K)QQVPSGESAILDR(V)	99%	52.3	41.5	37.2	2		701.22	1,400.43	2	1.7	-210	3680		0.0000	270	282	0		File:P073910....
☑	✓	(K)QQVPSGESAILDR(V)	96%	46.4	41.4	33.1	2		701.17	1,400.33	2	1.6	-280	3770		0.0000	270	282	0		File:P073910....
☑	✓	(R)VVSEDFLQDVSASTK(S)	100%	122.8	39.8	102.7	2		813.16	1,624.31	2	0.51	-310	4450		0.0000	453	467	0		File:P073910....
☑	✓	(R)VVSEDFLQDVSASTK(S)	100%	71.8	40.1	65.9	2		813.72	1,625.43	2	1.6	-230	4420		0.0000	453	467	0		File:P073910....
☑	✓	(K)AEPVEVVAPR(G)	93%	42.9	41.4	14.9	2		534.15	1,066.29	2	0.70	-280	3470		0.0000	487	496	0		File:P073910....
☑	✓	(K)VFSATLGLVDIVK(G)	100%	65.1	41.6	38.8	2		681.78	1,361.55	2	0.75	-190	5060		0.0000	552	564	0		File:P073910....
☑	✓	(K)VFSATLGLVDIVK(G)	98%	51.2	40.5	35.3	2		682.07	1,362.13	2	1.3	240	5030		0.0000	552	564	0		File:P073910....
☑	✓	(R)VGTVIGSNK(L)	93%	42.8	42.9	22.4	2		438.11	874.21	2	0.71	-330	3140		0.0000	592	600	0		File:P073910....
☑	✓	(K)AMVEYEIDLQK(M)	100%	80.2	40.2	58.6	2	Oxidation (+16)	678.06	1,354.11	2	0.46	340	3990		0.0000	685	695	0		File:P073910....
☑	✓	(K)AMVEYEIDLQK(M)	100%	67.3	39.9	40.6	2	Oxidation (+16)	678.01	1,354.01	2	0.36	260	3960		0.0000	685	695	0		File:P073910....
☑	✓	(K)AMVEYEIDLQK(M)	93%	42.9	39.8	3.3	2	Oxidation (+16)	678.02	1,354.03	2	0.38	280	3930		0.0000	685	695	0		File:P073910....
☑	✓	(K)KPPLLNNADSVQAK(V)	94%	43.8	40.1	28.5	2		748.64	1,495.27	2	1.4	300	4360		0.0000	748	761	0		File:P073910....
☑	✓	(K)KPPLLNNADSVQAK(V)	93%	50.5	41.2	30.9	2		499.32	1,494.94	3	1.1	77	4390		0.0000	748	761	0		File:P073910....
☑	✓	(R)TTNFAGILSQGLR(I)	100%	84.4	41.5	56.7	2		690.21	1,378.41	2	1.7	-250	4820		0.0000	866	878	0		File:P073910....
☑	✓	(R)TTNFAGILSQGLR(I)	100%	70.6	41.6	47.4	2		689.90	1,377.79	2	1.0	30	4850		0.0000	866	878	0		File:P073910....
☑	✓	(K)GIYFADMVSK(S)	99%	54.7	41.9	39.4	2	Oxidation (+16)	574.10	1,146.19	2	0.64	-310	4150		0.0000	894	903	0		File:P073910....
☑	✓	(K)GIYFADMVSK(S)	98%	49.9	41.9	30.4	2	Oxidation (+16)	574.11	1,146.21	2	0.66	-300	4180		0.0000	894	903	0		File:P073910....

Appendix Table S2

Characteristics of 183 breast invasive ductal carcinoma patients

Characteristics	Breast cancer (n=183)	
	n	percent
**Age (range between 19 and 87, mean:49)**		
<50	106	57.9%
≥50	77	42.1%
**Histological grade**		
Moderate differentiation	124	67.8%
Poor differentiation	58	31.7%
**LN metastasis**		
N0	114	62.3%
N1-3	69	37.7%
**T stage**		
T1	11	6.01%
T2	120	65.6%
T3	32	17.5%
T4	20	10.9%
**Clinical stage**		
I	10	5.5%
II	130	71.0%
III	42	22.9%
**ER Status**		
ER -	92	50.3%
ER +	83	45.4%
**PR Status**		
PR -	119	65.0%
PR +	58	31.7%
**HER2 Status**		
HER 2 -	115	59.9%
HER2 +	62	33.9%

Appendix Table S3

Characteristics of breast invasive ductal carcinoma patients with prognosis data

	Breast cancer (n=117)	
**Characteristics**	**n**	**percent**
**Age (range between 19 and 87, mean:49)**		
<50	38	31.6 %
≥50	81	68.4 %
**LN metastasis**		
N0	66	56.4 %
N1-3	53	43.6 %
**T stage**		
T1	47	40.2 %
T2	70	58.1 %
T3	2	1.70 %
**Clinical stage**		
II	78	65.0 %
III	41	35.0 %
**ER Status**		
ER -	35	29.9 %
ER +	81	67.5 %
**PR Status**		
PR -	58	48.7 %
PR +	59	49.6 %
**HER2 Status**		
HER 2 -	93	78.6 %
HER2 +	24	19.7 %

Appendix Table S4: exact p-Values

Figure panel	Comparison	*p*-value
**Figure 7H**	Olaparib 0.3µM: $KLF4^{+/+}$ vs $KLF4^{-/-}$	8.32E-04
	Olaparib 1.0µM: $KLF4^{+/+}$ vs $KLF4^{-/-}$	1.88E-04
	Olaparib 3.0µM: $KLF4^{+/+}$ vs $KLF4^{-/-}$	1.03E-03
	Olaparib 10.0µM: $KLF4^{+/+}$ vs $KLF4^{-/-}$	1.53E-02
**Figure 7I**	Olaparib 0.3µM: $KLF4^{WT}$ vs $KLF4^{YYR/AAA}$	2.23E-03
	Olaparib 1.0µM: $KLF4^{WT}$ vs $KLF4^{YYR/AAA}$	5.61E-03
	Olaparib 3.0µM: $KLF4^{WT}$ vs $KLF4^{YYR/AAA}$	0.0439
	Olaparib 10.0µM: $KLF4^{WT}$ vs $KLF4^{YYR/AAA}$	2.29E-04
**Figure 7K**	Olaparib 1.0µM: $KLF4^{WT}$ vs $KLF4^{YYR/AAA}$	0.0287
	Olaparib 3.0µM: $KLF4^{WT}$ vs $KLF4^{YYR/AAA}$	0.0191
	Olaparib 10.0µM: $KLF4^{WT}$ vs $KLF4^{YYR/AAA}$	8.72E-03
	Rucaparib 1.0µM: $KLF4^{WT}$ vs $KLF4^{YYR/AAA}$	5.64E-03
	Rucaparib 10.0µM: $KLF4^{WT}$ vs $KLF4^{YYR/AAA}$	4.65E-06
	Niraparib 1.0µM: $KLF4^{WT}$ vs $KLF4^{YYR/AAA}$	6.87E-05
	Niraparib10.0µM: $KLF4^{WT}$ vs $KLF4^{YYR/AAA}$	4.68E-06
**Figure 7L**	Olaparib 1.0µM: Vector vs $KLF4^{YYR/AAA}$	4.32E-03
	Olaparib 3.0µM: Vector vs $KLF4^{YYR/AAA}$	0.0165
**Figure 7M**	Olaparib 1.0µM: Vector vs $KLF4^{YYR/AAA}$	5.08E-03
	Olaparib 3.0µM: Vector vs $KLF4^{YYR/AAA}$	6.93E-03
	Olaparib 10.0µM: Vector vs $KLF4^{YYR/AAA}$	8.74E-05
**Figure 7N**	Olaparib 0.1µM: Vector vs $KLF4^{YYR/AAA}$	9.12E-05
	Olaparib 0.3µM: Vector vs $KLF4^{YYR/AAA}$	1.74E-06
	Olaparib 1.0µM: Vector vs $KLF4^{YYR/AAA}$	2.36E-06
**Figure 7O**	Olaparib 0.1µM: ShKLF4 vs Vector	3.60E-04
	Olaparib 1.0µM: ShKLF4 vs Vector	0.0147
	Olaparib 10µM: ShKLF4 vs Vector	2.61E-03
**Figure 7P**	Olaparib 0.1µM: ShKLF4 vs Vector	0.023
	Olaparib 1.0µM: ShKLF4 vs Vector	5.34E-04
**Figure 7Q**	Doxorubicin 0.01µM: ShKLF4 vs Vector	3.20E-03
	Doxorubicin 0.05µM: ShKLF4 vs Vector	1.78E-03
	Doxorubicin 0.2µM: $KLF4^{WT}$ vs Vector	1.34E-04
	Doxorubicin 1.0µM: $KLF4^{WT}$vs Vector	3.63E-04
**Figure 7R**	CDDP 2.5µM: ShKLF4 vs Vector	0.0233
	CDDP 10µM: ShKLF4 vs Vector	7.21E-03
	CDDP 40µM: ShKLF4 vs Vector	4.53E-04
**Appendix Figure S2G**	24h: $KLF4^{+/+}$ vs $KLF4^{-/-}$	3.17E-04
**Appendix Figure S2H**	0h: $KLF4^{+/+}$ vs $KLF4^{-/-}$	2.76E-03
	24h: $KLF4^{+/+}$ vs $KLF4^{-/-}$	6.23E-05

**Appendix Figure S2I**	0h: $KLF4^{+/+}$ vs $KLF4^{-/-}$	1.43E-06
	24h: $KLF4^{+/+}$ vs $KLF4^{-/-}$	7.23E-05
	48h: $KLF4^{+/+}$ vs $KLF4^{-/-}$	3.80E-04
**Appendix Figure S2J**	0h: $KLF4^{+/+}$ vs $KLF4^{-/-}$	0.0238
	4h: $KLF4^{+/+}$ vs $KLF4^{-/-}$	5.02E-04
	24h: $KLF4^{+/+}$ vs $KLF4^{-/-}$	2.37E-03
**Appendix Figure S2K**	Control: I-SCE vs KLF4 KD	1.43E-03
	Radiation: I-SCE vs KLF4 KD	5.81E-04
**Appendix Figure S5I**	Dox: ShScr vs SHKLF4	3.05E-03
	Dox: ShScr vs KLF4	4.90E-04
	Ola: ShScr vs KLF4	2.08E-05
	Dox/Ola: ShScr vs KLF4	3.92E-06
**Appendix Figure S5J**	Vehicle: KLF4WT vs Vector	1.77E-06
	Vehicle: $KLF4^{WT}$ vs $KLF4^{YYR/AAA}$	4.09E-07
	Olaparib: KLF4WT vs Vector	4.82E-03
	Olaparib: $KLF4^{WT}$ vs $KLF4^{YYR/AAA}$	0.0431
