# Supplementary figures and images for: New insight into the significance of KLF4 PARylation in genome stability, carcinogenesis, and therapy
Source: EMBO Mol Med. 2020 Nov 24;12(12):e12391. doi: 10.15252/emmm.202012391 (PMC7721363; doi:10.15252/emmm.202012391)

Appendix Figure S5

A

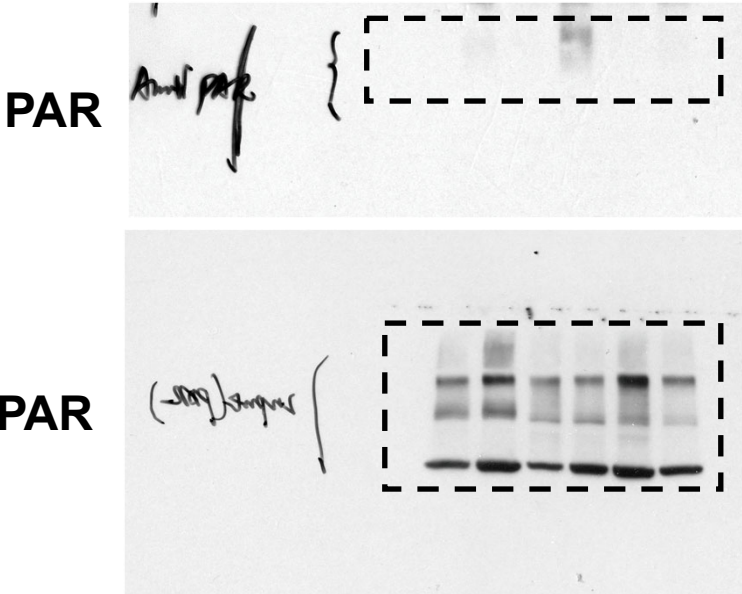

B

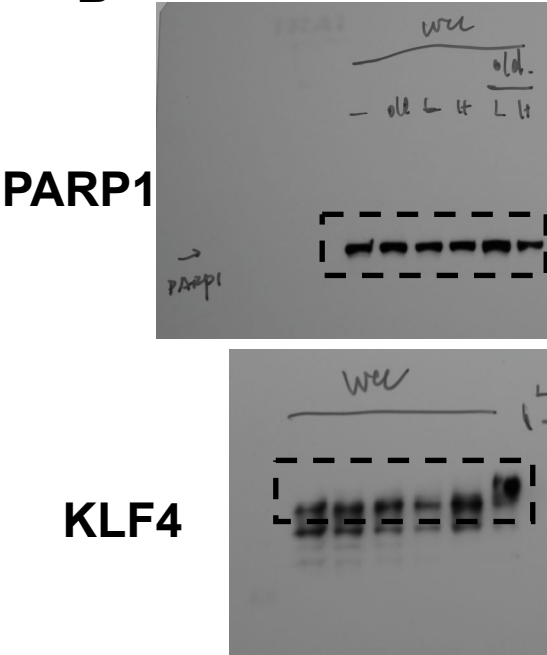

D

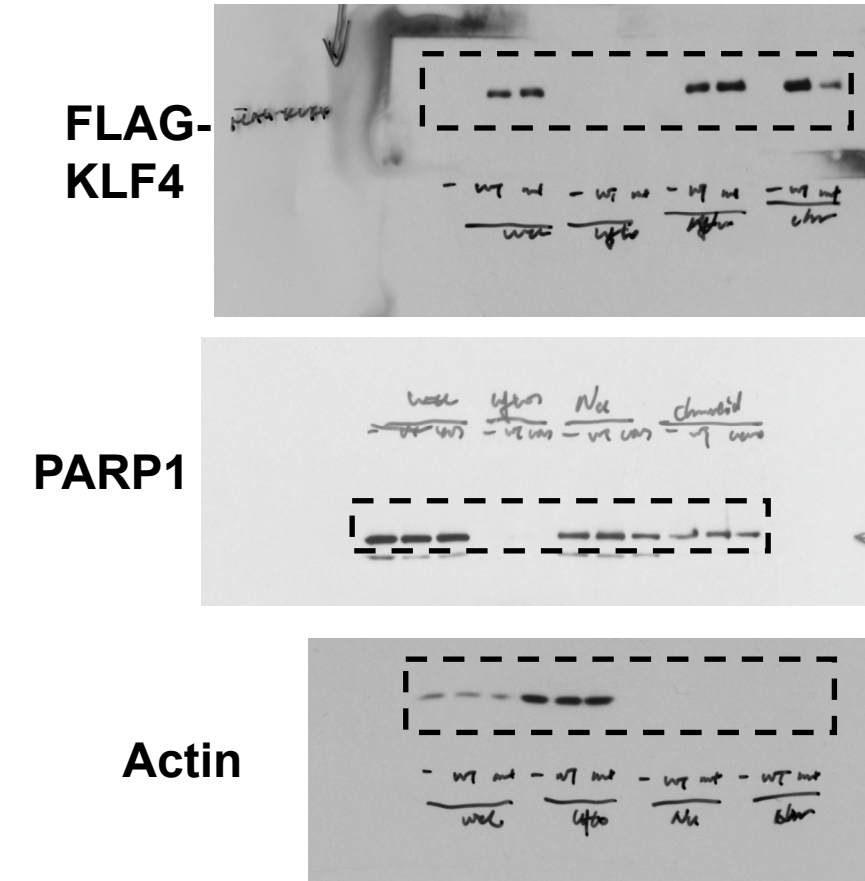

Actin

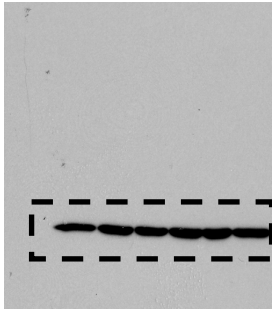

PARP1

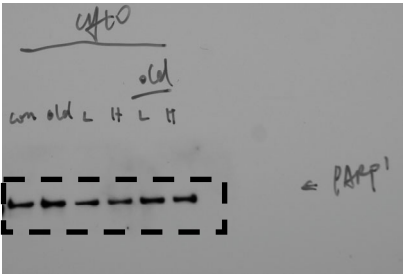

Actin

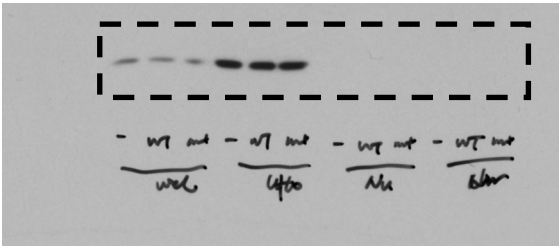

KLF4

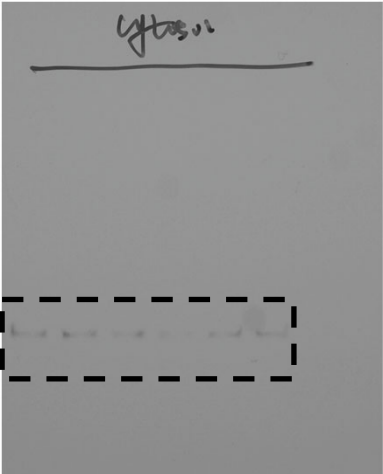

Actin

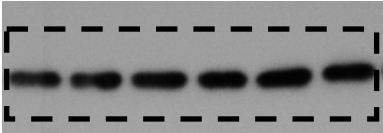

## Appendix Figure S5

**E**

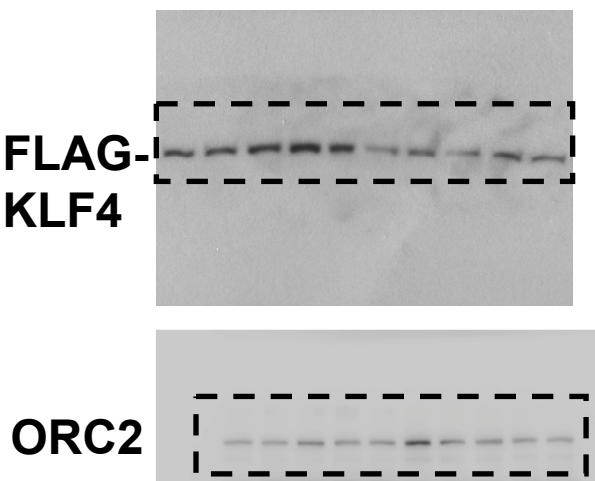

**F**

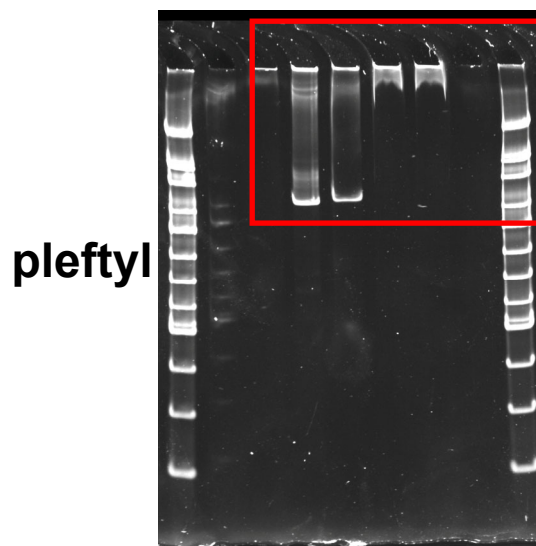

**G**

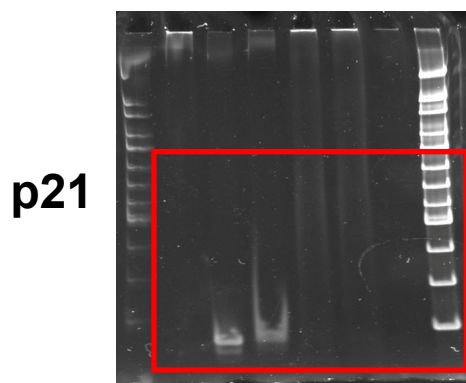

**H**

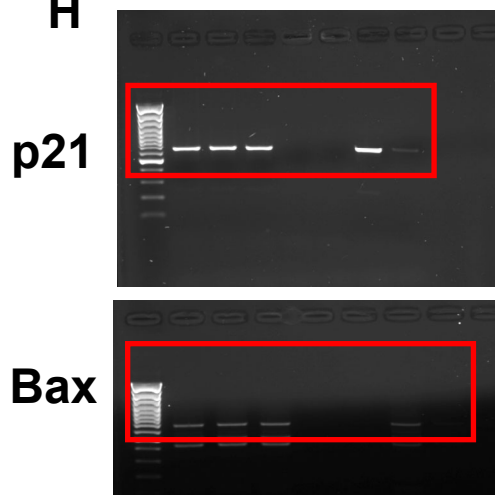

Supplement: Supplementary file 2 — Source Data for Appendix [file EMMM-12-e12391-s002.pdf]
